# Assessment of utilization of provider-initiated HIV testing and counseling as an intervention for prevention of mother to child transmission of HIV and associated factors among pregnant women in Gondar town, North West Ethiopia

**DOI:** 10.1186/1471-2458-12-226

**Published:** 2012-05-11

**Authors:** Marelign Tilahun Malaju, Getu Degu Alene

**Affiliations:** 1 Department of Public Health, College of Medicine and Health Sciences, Arba-Minch University, P.O. Box: 21, Arba-Minch, Ethiopia; 2 School of Public Health, College of Medicine and Health Sciences, University of Gondar, P.O. Box: 196, Gondar, Ethiopia

## Abstract

**Background:**

Detection of maternal HIV infection early in pregnancy is critical for prevention of mother to child transmission of HIV/AIDS. Most efforts have focused on VCT as the primary means of encouraging people to become aware of their HIV status. However, its uptake is low in many parts of sub-Saharan Africa including Ethiopia. Provider-initiated HIV testing and counseling provides a critical opportunity to diagnose HIV infection, to begin chronic care, and to prevent mother to child transmission. However, little is known about its acceptance and associated factors among pregnant women in the country and particularly in the present study area.

**Methods:**

Health institution based cross-sectional quantitative study was conducted in Gondar town from July 22-August 18, 2010. A total of 400 pregnant women were involved in the study using stratified sampling technique and multiple logistic regression analysis was employed using SPSS version 16.

**Results:**

A total of 400 pregnant women actively participated in this study and 330 (82.5%) of them accepted provider-initiated HIV testing and counseling to be tested for HIV and 70(17.5%) of them refused. Acceptance of provider-initiated HIV testing and counseling was positively associated with greater number of antenatal care visits [Adj. OR (95%CI)=2.64(1.17, 5.95)], residing in the urban areas[Adj. OR (95%CI)=2.85(1.10, 7.41)], having comprehensive knowledge on HIV [Adj. OR (95%CI)=4.30(1.72, 10.73)], positive partners reaction for HIV positive result [Adj. OR (95%CI)=8.19(3.57, 18.80)] and having knowledge on prevention of mother to child transmission of HIV[Adj. OR (95%CI)=3.27(1.34, 7.94)], but negatively associated with increased maternal age and education level.

**Conclusion:**

Utilization of provider-initiated HIV testing and counseling during antenatal care was relatively high among pregnant women in Gondar town. Couple counseling and HIV testing should be strengthened to promote provider-initiated HIV testing and counseling among male partners and to reduce HIV related violence of women from their partner and access to and consistent use of antenatal care should be improved to increase the uptake of provider-initiated HIV testing and counseling service.

## Background

Worldwide, HIV/AIDS poses an enormous challenge on the survival of mankind. In 2008, over 33.4 million people were living with the virus and of this 67% were in sub Saharan Africa. Annually 430,000 children are infected with HIV; mainly due to mother to child transmission and 90% of this occurs in Sub-Saharan Africa [1].

In 2009 the prevalence of HIV in Ethiopia was estimated at 2.3% with differentials: urban (7.7%), rural (0.9%), male (1.8%), and female (2.8%). The number of people living with HIV/AIDS was 1,116,216 of which 84,189 were pregnant women, 72,945 were children under 15years and annual HIV positive births were 14,140. In Amhara region, northwest Ethiopia the prevalence of HIV was 2.8%. The prevalence among males and females were 2.2% and 3.4% respectively and the annual HIV positive births were 5,030 [2].

In the absence of any intervention of mother to child transmission of HIV(i.e. exclusive breast feeding and use of antiretroviral drugs), the risk of a baby acquiring the virus from an infected mother ranges from 15% to 25% in industrialized countries, and 25% to 35% in developing countries. HIV transmission rate and time of transmission is estimated to be 5% to 10% during pregnancy, 10% to 15% during delivery, and 5% to 20% during breast feeding [3-5].

Detection of maternal HIV infection early in pregnancy provides a gateway to prevention of mother to child transmission (PMTCT) of HIV [6]. Most efforts have focused on voluntary counseling and testing (VCT) as the primary means of providing testing and encouraging people to become aware of their HIV status. However, there has been wide spread concern about the slow uptake of VCT in many parts of sub-Saharan Africa [7,8] with the number being tested far fewer than that required to identify even those requiring highly active antiretroviral therapy (HAART) [9,10].

In Ethiopia, from the 2005 Demographic and Health survey (DHS), only 6% of men and 4% of women were reported being tested nationally and 1.8% women in Amhara region, northwest Ethiopia [11]. Nationally only 8% of HIV infected pregnant women have received antiretroviral (ARV) drugs to reduce the risk of mother to child transmission (MTCT) of HIV/AIDS during 2009 [12]. As part of the response to the problem, the World Health Organization (WHO) has introduced provider-initiated HIV testing and counseling (PITC) approach [13] and subsequently, the revised version of the Ethiopian Prevention of Mother to Child transmission of HIV (PMTCT) guideline issued in 2007 recommends provider initiated HIV testing and counseling (PITC) as a routine care for pregnant women in Ante natal Care (ANC) clinics to decrease mother to child transmission (MTCT) of HIV/AIDS [14]. However, the acceptance rate of provider initiated HIV testing and counseling and factors contributing its utilization has not been well studied in Ethiopia. From July 22 August 18, 2010 we conducted this study to assess acceptance rate of provider initiated HIV testing and counseling (PITC) as an intervention for prevention of mother to child transmission (PMTCT) of HIV and factors associated with its acceptance among pregnant women in Health facilities of Gondar Town, Northern Ethiopia.

## Methods

Health institution based cross-sectional quantitative study was conducted from July 22 August 18, 2010. The study was conducted in Gondar town which is located about 750km northwest from Addis Ababa the capital city of Ethiopia. The town has 12 administrations and a population of 220,184 and of this 51,963 was females of reproductive age group (1549years) with in the area of 41.27 square Km. There is one referral hospital and five health centers which offer ANC, PITC and PMTCT services in the town. The source population was all pregnant women attending antenatal care in public health facilities since these health facilities serve the majority of the population in ANC service especially the rural and poor population. The study population was all pregnant women attending antenatal care during the data collection period in public health facilities of Gondar town, Northwest Ethiopia.

### The antenatal care services

Antenatal care services for all pregnant women include: At least four focused antenatal care visits (1st as early in pregnancy as possible, 2nd at 2832weeks, 3rd after 36weeks, and 4th before expected date of delivery or when woman needs to consult), Routine laboratory diagnostic tests (hemoglobin, syphilis, HIV, glucose, and blood pressure), Tetanus toxoid vaccination, Malaria prevention and treatment, Infant feeding counseling with emphasis on exclusive breastfeeding for the first six months and counseling on danger signs of obstetric complications.

### The PMTCT program

The Government of the Federal Democratic Republic of Ethiopia is committed to reduce the spread of HIV/AIDS and address the consequences of the epidemic in the population. The national HIV/AIDS policy was enacted in 1998; and in 2001, the National HIV/AIDS Council declared HIV a national emergency. The National HIV/AIDS strategic framework calls for a multi-sectorial response, guaranteeing rights of all people living with HIV/AIDS, and facilitating the supply and use of antiretroviral drugs. Ethiopia has adopted the WHO/UNICEF/UNAIDS 4-pronged PMTCT strategy as a key entry point to HIV care for women, men and families. Prevention of mother-to-child transmission services began in 2003, but suffers from low utilization of antenatal care and delivery services; and only 0.8% of HIV infections among births to HIV positive mothers were averted in 2005/6 through PMTCT programs [14].

### Sampling procedure

Five health centers and one hospital which offer ANC, PITC and PMTCT were included in the study. Stratified sampling technique was used to select the study units in each health institution. Based on the number of customers who visited each health institution during the previous ten months (monthly report of each health institution), proportional allocation of the total sample size was carried out to attain the required sample size in each health institution. Finally, the determined sample for each health institution was achieved through exit interview from systematically sampled and voluntarily consenting pregnant women with in four weeks of working days. Pregnant women attending antenatal care in health institutions of Gondar Town, Northwest Ethiopia during the data collection period was included in the study. All pregnant women who are unable to communicate (having hearing problem and unable to communicate with sign languages) were excluded from the study.

### Data collection and quality control

Clinic staff who provided pretest counseling (primarily dedicated PMTCT counselors with more than 1year of experience) were trained to conduct PITC sessions and were provided with scripts on how to introduce the HIV test as part of a package of routine antenatal services including data collection and interview techniques. The PITC session included basic information about HIV transmission, PMTCT, and ARV therapy; a brief explanation of all tests done during ANC (hemoglobin, syphilis, HIV, glucose, and blood pressure); and a statement that all tests are routine but that patients have the right to refuse tests they do not want.

A structured questionnaire which had been previously pre-tested and subsequently finalized after modification was used to elicit the following information from the study participants: socio-demographic data, knowledge of PMTCT of HIV, acceptability of PITC, number of antenatal care visits, comprehensive knowledge on HIV/AIDS, attitude towards PITC, risk perception of HIV, perceived benefit of HIV test, attitude towards counselors, partners reaction for HIV positive test result and stigmatizing attitude towards people having HIV/AIDS. The completeness and consistency of data was established through direct and daily supervision by the supervisor and principal investigator. Data coding, cleaning and verification were performed to assure quality of data.

### Data processing and analysis

Sample size was determined using the formula of a single population proportion estimation and calculated using software Epi-info stat calc. by taking 59% proportion, 5% of absolute precision and with 95% confidence interval. Non-response rate in this study was estimated to be 10% i.e. 38, and hence an overall sample size of 410 Pregnant women were recruited in the study.

Data were entered and analyzed using SPSS software version 16. Descriptive statistics such as frequencies and proportion was used to describe the study population in relation to relevant variables. Explanatory variables found to be statistically significant in bivariate logistic regression analysis were entered into multiple logistic regression analysis (backward stepwise method) for adjustment of confounders. Odds ratio, confidence interval and P-value were computed to assess the presence and degree of association between dependent and independent variables.

### Ethical consideration

Ethical clearance to conduct the study was obtained from Ethical Review Board, School of public health, University of Gondar and permission to conduct the study in each health facilities was secured from the respective Health institutions in Gondar Town. Verbal informed consent from each study participants was obtained after clear explanation about the purpose of the study.

## Results

From the total 410 pregnant women recruited 400 participated actively in this study in health facilities of Gondar town making the response rate of 97.6%.

About 285 (71.2%) of the study population (65.5% acceptors & 5.7% non-acceptors of PITC) were from urban areas while the rest 115 (28.8%) (17% of acceptors & 11.8% of non-acceptors of PITC) were from rural part of the study area. Over 90.0 %( 361) of the respondents were married followed by those who were divorced/separated/widowed 20(5.0%) and the majority 194(48.5%) of them had no education, followed by those who attended school secondary and above education 168(42.0%).

The most frequent occupation was housewife (59.0%) seconded by government employed (16.0%) and merchant (10.2%), respectively. The majority 391 (97.8%) of the study participants were Amhara by ethnicity (80.8%acceptors & 17.0% non-acceptors) followed by Tigre and Gurage.

Most of the study participants 361 (90.2%) were followers of orthodox Christianity (74.2% acceptors & 16.0% non-acceptors of PITC) followed by Muslim 35(8.8%). Regarding their age distribution 184(45.8%) of them were in the age range between 2534years with mean (SD) age of 25.37(5.25) and majority (48.0%) of them (42.0% acceptors & 6.0% non-acceptors) have monthly expenditure of 451999 birr per month. Among the study participants 330 (82.5%) of them were acceptors of provider initiated HIV testing and counseling (Table 1).

**Table 1:** Socio-demographic Characteristics of Acceptors and Non-Acceptors of PITC among pregnant women attending ANC in Health Facilities of Gondar Town, Gondar, 2010

**Variables**	**Acceptors (330)**	**Non-acceptors (70)**	**Total (400)**
**Age**[Mean(SD)=25.37 (5.25)]			
1524	154(38.5)	20(5.0)	174(43.4)
2534	154(38.5)	29(7.2)	183(45.8)
35-49	22(5.5)	21(5.2)	43(10.8)
**Residence**			
Urban	262(65.5)	23(5.8)	285(71.2)
Rural	68(17.0)	47(11.8)	115(28.8)
**Ethnic group**			
Amhara	323(80.8)	68(17.0)	391(97.8)
Tigray	6(1.5)	2(0.5)	8(2.0)
Gurage	1(0.2)	0(0.0)	1(0.2)
**Religion**			
Orthodox Christian	297(74.2)	64(16.0)	361(90.2)
Muslim	29(7.2)	6(1.5)	35(8.8)
Protestant	4(1.0)	0(.0)	4(1.0)
**Education**			
No education	144(36.0)	50(12.5)	194(48.5)
Primary Education	30(7.5)	8(2.0)	38(9.5)
Secondary & above	156(39.0)	12(3.0)	168(42.0)
**Occupation**			
Government employed	61(15.2)	3 (0.8)	64(16.0)
Merchant	33 (8.2)	8 (2.0)	41(10.2)
House wife	183(45.8)	53(13.2)	236(59.0)
Student	35(8.8)	2 (0.5)	37(9.2)
Others*	18(4.5)	4 (1.0)	22(5.5)
**Marital Status**			
Never married	18(4.5)	1 (0.2)	19(4.8)
Married/living together	298 (74.5)	63 (15.8)	361(90.2)
Divorced/separated/widowed	14(3.5)	6 (1.5)	20(5.0)
**Monthly Expenditure**			
450 birr/month	71 (17.8)	36 (9.0)	107(26.8)
451999 birr/month	168 (42.0)	24 (6.0)	192(48.0)
1000 birr/month	91 (22.8)	10 (2.5)	101(25.2)

* Bartender, daily laborer and Jobless

### Knowledge and attitude of respondents towards PITC among pregnant women attending ANC in Health facilities of Gondar town

Majority of the respondents in this study (97.3% acceptors, 94.3% non-acceptors and overall 96.8% of mothers) heard about the existence of provider-initiated HIV testing and counseling service during pregnancy and 91.2% acceptors, 90.0% non-acceptors and overall 91.0% of the respondents were in favor of provider-initiated HIV testing and counseling.

Regarding to specific attitudes towards PITC, 46.7% of acceptors and 32.9% of non-acceptors agreed on routine testing makes easier for ANC clients to get tested for HIV and 40.3% of acceptors and 31.4% of non-acceptors agreed on PITC helps ANC clients to take ARV drugs to prevent a baby from HIV.

On the other hand 12.7% acceptors, 41.4% non-acceptors and overall 17.75% of mothers believed that routine testing would cause people to avoid seeing their health care provider for fear of being tested and 4.5% acceptors, 25.7% non-acceptors and overall 8.25% of mothers thought that routine testing would lead to more violence against women (Table 2).

**Table 2:** Knowledge and Attitude Related to PIHCT among pregnant Women attending ANC in Health facilities of Gondar Town, Gondar, Northwest Ethiopia 2010

**Questions**	**Acceptors n=330**	**Non-acceptors n=70**	**Total n=400**
**Ever heard of PITC**	321 (97.3)	66 (94.3)	387(96.8)
Yes	9 (2.7)	4 (5.7)	13 (3.2)
No			
**Sources of information:**	**n=321**	**n=66**	**n=387**
Health workers	225 (70.1)	45 (68.2)	270(70.0)
Family members	32 (10.0)	8 (12.1)	40(10.3)
Mass media	6 (2.0)	6 (9.1)	12(3.1)
Friends	3 (1.0)	2 (3.0)	5(1.3)
Multiple answers	54 (16.9)	5 (7.6)	59(15.3)
**In favor of PITC**	**n=330**	**n=70**	**n=400**
Yes	301 (91.2)	63 (90.0)	364(91.0)
No	29 (8.8)	7 (10.0)	36(9.0)
**Specific Attitudes on PITC**			
**Those women who agree that PITC:**	**n=330**	**n=70**	**n=400**
Makes easier for ANC clients to get tested.	154 (46.7)	23(32.9)	177(44.25)
Avoid discrimination of HIV positive women	32 (9.7)	8 (11.4)	40(10.0)
Avoids violence towards women.	27 (8.2)	5 (7.1)	32(8.0)
Helps ANC clients to get an access for ART.	84 (25.5)	13 (18.6)	97(24.25)
Increase number of HIV tested pregnant women.	43 (13.0)	4 (5.7)	47(11.75)
Helps to take ARV drugs to prevent a baby from HIV.	133 (40.3)	22 (31.4)	155(38.75)
Helps to decide on what to feed a baby to prevent from HIV.	68 (20.6)	4 (5.7)	72(18.0)
Leads people to avoid going to a health facility for fear of HIV testing.	42 (12.7)	29 (41.4)	71(17.75)
Increase violence towards women.	15 (4.5)	18 (25.7)	33(8.25)
Violet ANC clients human right.	12 (3.6)	3 (4.3)	15(3.75)
Leads to more discrimination of HIV positive women.	9 (2.7)	3 (4.3)	12(3.0)

### Reasons for acceptance and refusal of PITC among pregnant women attending ANC in health facilities of Gondar town

The most frequent reasons given for accepting provider-initiated HIV testing and counseling were concern for their own health and to protect their children (44% and 35% respectively). The major barriers for acceptance of provider-initiated HIV testing and counseling were fear of partners reaction for HIV test (31.4%), was not ready for HIV test (14.3%), need for partners consent (10%), afraid to know if they were HIV-positive and not being sure of the confidentiality of the test (8.6%). (Table 3)

**Table 3:** reasons for acceptance and refusal of PITC among pregnant women attending ANC in Health facilities of Gondar town, Gondar, Northwest Ethiopia, 2010

**Reasons for Acceptance of PITC:**	**n=330**
To protect my child from HIV	114(35.0)
To protect my partner from HIV	4(1.0)
To know my HIV status	147(44.0)
Multiple answers	65(20.0)
**Reasons for Refusal of PITC:**	**n=70**
Fear of stigma and discrimination	2(2.9)
Fear of partners reaction for HIV positive result	22(31.4)
Fear of knowing HIV positive test result	6(8.6)
Lack of HIV risk perception	5(7.1)
Not being sure of HIV test confidentiality	6(8.6)
Need for partners consent	7(10.0)
Was not ready for HIV test	10(14.3)
Multiple answers	12(17.2)

### Association between acceptance of provider-initiated HIV testing and counseling and each explanatory variable

In order to measure the association between acceptance of PITC and a number of explanatory variables, crude OR and adjusted OR with 95% CI were employed.

After controlling for confounders, the association between selected explanatory variables and acceptability of PITC is presented in Table 4.

**Table 4:** Association between acceptance of provider-initiated HIV testing and counseling and each explanatory variable (Crude & adjusted OR)

**Explanatory Variable**	**Acceptance of PITC**		**Crude OR (95% CI)**	**Adjusted OR (95% CI)**	**P- value**
	**Yes(1)**	**No(0)**			
**Age**					
1524	154	20	7.35(3.44, 15.69)	5.55(1.57, 19.66)	0.024
2534	154	29	5.07(2.47, 10.39)	3.87(1.23, 12.15)	
35-49	22	21	1.00	1.00	
**Residence**					
Urban	262	23	7.87(4.47, 13.86)	2.85(1.10, 7.41)	0.031
Rural	68	47	1.00	1.00	
**Education level**					
No Education	144	50	0.22(0.11, 0.43)	3.64(1.04, 12.78)	0.067
Primary education	30	8	0.29(0.11, 0.77)	1.03(0.25, 4.35)	
Secondary & above	156	12	1.00	1.00	
**Monthly Expenditure**					
1000 birr/month	91	10	4.61(2.14, 9.93)	2.87(1.11, 7.44)	0.078
451999 birr/ month	168	24	3.55(1.98, 6.38)	1.34(0.41, 4.32)	
450 birr/month	71	36	1.00	1.00	
**No of ANC visits**					
Two and above	240	23	5.45(3.13, 9.49)	2.64(1.17, 5.95)	0.02
One	90	47	1.00	1.00	
**Have Comprehensive Knowledge on HIV/AIDS**					
Yes	224	15	7.75(4.19, 14.35)	4.30(1.72, 10.73)	0.002
No	106	55	1.00	1.00	
**Attitude towards PITC**					
More favourable	267	14	16.95(8.88, 32.37)	6.17(2.59, 14.74)	<0.001
Less favourable	63	56	1.00	1.00	
**Perceived Partner's reaction for HIV positive result**					
Positive	271	15	16.84(8.91, 31.83)	8.19(3.57, 18.80)	<0.001
Negative	59	55	1.00	1.00	
**Have Knowledge on PMTCT**					
Yes	221	14	8.11(4.32, 15.21)	3.27(1.34, 7.94)	0.009
No	109	56	1.00	1.00	

For explanatory variables having more than two categories, the overall significance is given by their corresponding P-values

The assessment made whether the required assumptions for the application of multiple logistic regression was fulfilled showed that this parsimonious model adequately fits the data as P=0.551 (by using Hosmer and Lemeshow test)

Compared to older women (3549years), women aged 2534years were 3.9 times

[OR & (95%CI)=3.87(1.23, 12.15)] and women aged 1524years were 5.6 times

[OR & (95% CI)=5.55(1.57, 19.66)] more likely to accept PITC in the ANC clinics of Gondar town, northwest Ethiopia.

Compared to women who live in the rural areas, those women living in the urban areas were about 2.9 times [OR & (95%CI)=2.85 (1.10, 7.41)] more likely to accept PITC.

Women with monthly expenditure of 1000 birr per month were 2.9 times [OR & (95%CI)=2.87 (1.11, 7.44)] more likely to accept PITC than those with monthly expenditure of450 birr per month in ANC clinics of Gondar town health facilities.

Women who received two and above antenatal care during the current pregnancy were 2.6 times [OR & (95%CI)=2.64(1.17, 5.95)] more likely to accept PITC than those who attended antenatal care only once.

Compared to women who do not have comprehensive knowledge of HIV, women with good comprehensive knowledge of HIV were 4.3 times [OR & (95%CI)=4.30 (1.72, 10.73)] more likely to accept PITC in ANC clinics of Gondar, Northwest Ethiopia.

Women with more favourable attitude towards PITC were 6.2 times [OR & (95%CI)=6.17 (2.59, 14.74)] more likely to accept PITC than those women with less favourable attitude towards PITC.

Women who expect positive partners reaction for HIV positive result were 8.2 times [OR & (95%CI)=8.19(3.57, 18.80)] more likely to accept PITC than those who expect negative partners reaction for HIV positive result.

Compared to women with education level of secondary and above, those with no education were about 3.6 times [OR & (95%CI)=3.64(1.04, 12.78)] more likely to accept PITC in the ANC clinics of Gondar town, Northwest Ethiopia.

Finally those women with good knowledge of PMTCT were about 3.3 times [OR & (95%CI)=3.27(1.34, 7.94)] more likely to accept PITC than those who do not have PMTCT knowledge.

On the contrary, occupation, marital status, number of pregnancy, holding stigmatizing attitude towards people having HIV/AIDS, attitude towards counselors, availability & accessibility of health facilities with PITC service, perceived risk of acquiring HIV and perceived benefit of HIV testing were not independently associated with acceptability of PITC but were statistically significant in bivariate analysis.

## Discussion

The overall acceptance of PITC in this study was higher (82.5%) than the acceptance rates of VCT reported by different studies done in Illubabor, southwest Ethiopia (27.0%) and in Arba-Minch, southwest Ethiopia (74.4%) [15,16]. The significantly high uptake of PITC in this study could be related to multiple factors. Women were probably less fearful of participating in routine HIV testing because this approach would be perceived by her partner and family as standard of care offered to all ANC clients, thereby reducing the risk of stigma and other adverse social consequences when compared to the opt-in VCT policy. In addition, community sensitization, counseling sessions involving highly motivated PITC providers and availability of on-site rapid HIV testing may also have contributed to the significantly high HIV testing rates among pregnant women in this study.

There are differences in acceptability of PITC in different parts of Africa when compared with the acceptance of PITC in this study; 79% in two rural districts of Zimbabwe, 95% in Botswana, 88.3% in Cameroon, 99.9% in Urban Zimbabwe and 97% in rural Ugandan hospital [13,17-20].

Positive association was reported between acceptability of PITC during ANC in one hand, and having comprehensive knowledge on HIV, more favourable attitude towards PITC and knowledge on PMTCT on the other hand. One possible interpretation of the positive association between acceptance of PITC and having comprehensive knowledge on HIV, more favourable attitude towards PITC and knowledge on PMTCT is that those women who do not have comprehensive knowledge on HIV, more favourable attitude towards PITC and knowledge on PMTCT may fail to appreciate the importance of PITC and PMTCT and maternal and child health or may have less access to these services as well as to health education and promotion in general.

Greater number of ANC visits was positively associated with acceptance of PITC in this study and it is in agreement with the findings in two rural districts of Zimbabwe [13] but negatively associated in Cameroon [19]. The positive association between the number of ANC visits and acceptance of PITC could be that the less often a pregnant woman comes in contact with the health center, the less likely she is to hear about PMTCT, among other preventive messages and services. Improving access to and consistent use of ANC is therefore a high priority for improving PITC and PMTCT uptake as shown in Thailand [21].

This study has shown a negative association between maternal age and acceptance of PITC and this could be that younger and older women may differ in their perceived risk of HIV and understanding of the importance of HIV testing. This finding is in line with the findings in Cameroon and in two rural districts of Zimbabwe [9,19].

As to the perceived partners reaction for HIV positive result, non-acceptors of PITC in this study expect negative responses from their partner and this suggests a need to promote couple counseling and testing in the ANC clinics as recently shown in Uganda [17]. This finding is also in line with the findings from two rural districts of Zimbabwe [9].

When stratified by monthly expenditure, those women who had higher monthly expenditure (1000 birr per month) were more likely to accept PITC than those who had lower monthly expenditure (450 birr per month) and this could be that women with lower income are less able to make decisions on their own as they are less empowered economically.

Regarding association of education level with acceptance of PITC in this study, those women with no education were more likely to accept PITC than those with education level of secondary and above and this might be due to the fact that uneducated women might not refuse PITC because they believe that their doctor/nurse will react negatively to their refusal or they fear that they will receive inferior care as a result of their refusal or they might be denied their right to refuse PITC because socio-culturally uneducated women do not question the medical advice of their doctors or nurses and this possible explanation is in line with what has been expressed by many authors that proposing PITC would pose human rights challenges [22-24]. This possible explanation is also in line with the finding from Botswana where 68% of respondents felt that they could not refuse a test offered by their provider [25]. However, the overall association of education with acceptance of PITC in this study is marginally significant which indicates lack of power.

Concerning attitudes towards PITC in this study, 91% of them were in favor of PITC, 44.25% agreed that PITC makes easier for ANC clients to get tested and 38.8% agreed PITC helps to take ART to prevent a baby from HIV. While the percentage of those who are in favor of PITC (91%) is higher, those who agree on PITC makes easier for ANC clients to get tested and to take ART to prevent a baby from HIV were much lower than the finding from Botswana [25].

On the other hand, 17.8% of mothers in this study believed that PITC would cause people to avoid seeing their health care provider for fear of being tested and 8.25% of mothers thought that routine testing would lead to more violence against women and these percentages are lower when compared with the finding from population based study in Botswana [25].

The most frequent reasons for accepting PITC in this study were to protect their children (35%) and concern for their own health (44%) and the main reasons for refusal of PITC were fear of partners reaction for HIV test (31.4%), not ready for HIV test (14.3%) and afraid to know if they were HIV positive (8.6%) and these findings are consistent with the findings in urban Zimbabwe, Botswana and in two rural districts of Zimbabwe [9,20,25].

### Limitations

Our study had some potential limitations. Being a cross-sectional survey, causality cannot be inferred from our findings. The study is limited by being facility based and therefore eliminating pregnant women who did not patronize the services in the participating facilities. The findings therefore preclude generalization to all pregnant women in Gondar town, northwest Ethiopia indicating a need for further study of the acceptability of the opt-out test using a more representative sample of pregnant women in the country. We attempted to reduce social desirability bias by presenting study aims to the respondents in general terms but it might have been introduced because of self-report. Despite this limitation, the study provides useful information that will inform the implementation of PITC to PMTCT in Ethiopia.

## Conclusion

Our study findings have demonstrated that antenatal routine HIV counseling and testing seems to be largely acceptable to the pregnant women in northern Ethiopia and there is willingness to attend ANC even with the knowledge that HIV testing would be routinely offered. It is therefore being recommended that routine antenatal HIV testing should become the standard of care and be urgently implemented at all antenatal sites in Ethiopia. Given the high antenatal HIV prevalence, PITC will decrease the proportion of women who give birth with unknown HIV status and increase the number of mother-infant pairs who receive appropriate treatment for preventing mother-to-child transmission of HIV. All ANC staff should be trained as counselors and a system should be put in place to ensure, that every pregnant woman who comes to the ANC is offered the test. We encourage programs providing routine HIV testing to provide public education about the meaning of routine testing, ensure that patients are not tested without their knowledge, reassure patients that they can refuse testing and treatment they do not want, and raise awareness about the substantial benefits of HIV care and treatment.

## Authors' contributions

MT was investigator, involved in proposal writing, designing, and recruitment and training of supervisors and data collectors, analysis and write-up and in all stages of the project implementation. He did most of the analysis and write up of the paper. GD contributed in the designing of the methodology, lead investigator and involved in designing of project proposal, design of questionnaires, supervision and involved in giving comments in the progress of the project and final approval of the paper. All authors read and approved the final manuscript.

## Competing interests

The author(s) declare that they have no competing interests.

## Pre-publication history

The pre-publication history for this paper can be accessed here:

http://www.biomedcentral.com/1471-2458/12/226/prepub

## References

[B1] UNAIDS Report on the Global AIDS Epidemic 2009 UNAIDS, Geneva

[B2] Federal HIV/AIDS Prevention and Control Office, Federal Ministry of Health Single Point HIV prevalence Estimate 2007 Ethiopia

[B3] UNAIDS Prevention of HIV transmission from mother to child. Strategic options 1999 UNAIDS, Geneva, Switzerland

[B4] WHO HIV transmission through breast feeding. A review of available evidence 2004 World Health Organization, Geneva, Switzerland

[B5] Deneke K Rubin J Franklin N Guyon A Prevention of mother to child transmission (PMTCT) base line survey 2004 AED LINKAGES, Ethiopia

[B6] Bassett MT Ensuring a public health impact of programs to reduce HIV transmission from mothers to infants Am J Public Health 2002 92 347 351 11867305 10.2105/ajph.92.3.347 PMC1447074

[B7] World Health Organization (WHO) Leading the health sector response to HIV/AIDS 2003 World Health Organization, Geneva, Switzerland

[B8] The Voluntary HIV-1 Counseling and Testing Efficacy Study Group Efficacy of voluntary HIV-1 counseling and testing in individuals and couples in Kenya, Tanzania, and Trinidad Lancet 2000 356 103 112 10963246

[B9] Perez F Zvandaziva C Engelsmann B Dabis F Acceptability of routine HIV testing(opt-out) in antenatal services in two rural district of Zimbabwe J Acquir Immune Defic Syndr 2005 41 4 514 520 16652062 10.1097/01.qai.0000191285.70331.a0

[B10] Scott J Bansi L Ivens D HIV test uptake after introducing an opt-out screening system Int J STD AIDS 2006 17 213 16510018 10.1258/095646206775809277

[B11] Central Statistical Agency [Ethiopia] and ORC Macro Ethiopia Demographic and Health Survey 2005 2006 Central Statistical Agency and ORC Macro. Ethiopia, Addis Ababa, Ethiopia and Calverton, Maryland, USA

[B12] Federal HIV/AIDS prevention and control office Report on progress towards implementation of the UN Declaration of commitment on HIV/AIDS 2010 Federal HIV/AIDS Prevention and Control Office, Ethiopia

[B13] UNAIDS/WHO Guidance on Provider-Initiated HIV Testing and Counseling in health Facilities 2007 World Health Organization, Geneva, Switzerland http://www.who.int/hiv/who_pitc_guidelines.pdf Accessed May 31, 2010

[B14] MOH Guidelines for Prevention of Mother-to-Child Transmission of HIV in Ethiopia 2007 Federal HIV/AIDS Prevention and Control Office, Ethiopia

[B15] Kebede D Alemayehu A Biniam G Wassie L Yismaw D Uptake and barriers of voluntary counseling and testing among antenatal care attendants, south west Ethiopia Ethiopian Journal of Health Sciences 2006 16 1 71 81

[B16] Mesfin H Degu J Awareness of antenatal care clients on mother to child transmission (MTCT) of HIV infection and its prevention in Arbaminch, Ethiopia Ethiop J Heal Dev 2006 20 1 55 57

[B17] Homsy JH Kalamya JN Obonyo J Routine intra-partum HIV counseling and testing for prevention of mother-to-child transmission of HIV in a rural Ugandan hospital J Acquir Immune Defic Syndr 2006 42 2 149 154 16760796 10.1097/01.qai.0000225032.52766.c2

[B18] Creek TL Ntumy R Seipone K Successful introduction of routine opt-out HIV testing in antenatal care in Botswana J Acquir Immune Defic Syndr 2007 45 1 102 107 17460473 10.1097/QAI.0b013e318047df88

[B19] Kongnyuy E Mbu E Mbopi-Keou F Acceptability of intrapartum HIV counseling and testing in Cameroon BMC Pregnancy and Child Birth 2009 9 9 http://www.biomedcentral.com/1471-2393/9/9 10.1186/1471-2393-9-9 PMC2651846 19250517

[B20] Chandisarewa W Stranix-Chibanda L Chirapa E Routine offer of antenatal HIV testing (opt-out approach) to prevent mother-to-child transmission of HIV in urban Zimbabwe Bull World Health Organ 2007 85 11 843 850 18038074 10.2471/BLT.06.035188 PMC2636259

[B21] Teeraratkul A Simonds RJ Asavapiriyanont S Evaluating programs to prevent mother-to-child HIV transmission in two large Bangkok hospitals J Acquir Immune Defic Syndr 2005 38 2 208 212 15671807 10.1097/00126334-200502010-00013

[B22] McQuoid M Routine testing for HIV-ethical and legal implications S Afr Med J 2007 97 6 416 420 17691469

[B23] Csete J Elliot R Scaling up HIV testing: human rights and hidden costs HIV AIDS Policy Law Rev 2006 11 1 1 510 16805001

[B24] Rennie S Behets F The ethics of routine HIV testing in low income countries Bulletin of the WHO 2006 84 52 57 10.2471/blt.05.025536 PMC2626513 16501715

[B25] Weiser SD Heisler M Leiter K Routine HIV testing in Botswana: a population-based study on attitudes, practices and human rights concern PLoS Med 2006 3 7 261 10.1371/journal.pmed.0030261 PMC1502152 16834458

